# Ten Simple Rules for Graduate Students

**DOI:** 10.1371/journal.pcbi.0030229

**Published:** 2007-11-30

**Authors:** Jenny Gu, Philip E Bourne

Choosing to go to graduate school is a major life decision. Whether you have already made that decision or are about to, now it is time to consider how best to be a successful graduate student. Here are some thoughts from someone who holds these memories fresh in her mind (JG) and from someone who has had a whole career to reflect back on the decisions made in graduate school, both good and bad (PEB). These thoughts taken together, from former student and mentor, represent experiences spanning some 25 or more years. For ease, these experiences are presented as ten simple rules, in approximate order of priority as defined by a number of graduate students we have consulted here in the US; but we hope the rules are more globally applicable, even though length, method of evaluation, and institutional structure of graduate education varies widely. These rules are intended as a companion to earlier editorials covering other areas of professional development [1–7].

## Rule 1: Let Passion Be the Driving Force of Your Success

As with so many other things in life, your heart and then your head should dictate what thesis project makes sense to embark on. Doing your best work requires that you are passionate about what you are doing. Graduate school is an investment of up to a seven-year commitment, a significant chunk of your life. Use the time wisely. The educational system provides a variety of failsafe mechanisms depending on the part of the world where you study. Laboratory rotations and other forms of apprenticeship should not be overlooked, for they are opportunities to test the waters and measure your passion in a given subject area. It is also a chance to test your aptitude for research. Take advantage of it! Research is very different from simply taking courses. If you do not feel excited about doing research and the project selected, do not do it; reevaluate your career decisions.

## Rule 2: Select the Right Mentor, Project, and Laboratory

Finding the right mentor can be hard since it is not always possible to know the kind of mentoring that is going to work best for you until you actually start doing research. Some of us like to work independently, others like significant feedback and supervision. Talk to other students in the laboratory and get their impressions of how the principle investigator's mentoring works for them. In a large laboratory, chances are you will get less direct mentoring from the principle investigator. In that case, other senior scientists in the laboratory become important. What mentoring are they likely to offer? Judge, as best you can, if the overall environment will work for you. A key element is the standing of your mentor in his or her scientific field. When you graduate, the laboratory you graduate from is going to play a role in determining what opportunities exist for your postdoctoral work, either in academia, industry, or other sectors. Your proposed mentor should be very enthusiastic about the project you discuss. If he or she is not, you have the wrong mentor and/or project. At the same time, beware that such enthusiasm, however senior the mentor, may be misplaced as far as your interests are concerned. Gauge the novelty of the research project and potential for high-quality publications by doing your own background check through reading previously published research and talking to other scientists in related areas. Also consider if the project can be reasonably completed in the allocated time for graduation. To propel your career, you want to come out of a higher degree as a recognized individual having made a significant scientific contribution. Thus, it is absolutely critical that you do take the time to find the project and mentor that is going to fulfill this goal.

## Rule 3: Independent Thinking Is a Mark of a True Scientist

Regardless of your initial work habits and how much you depend on your mentor (Rule 2), eventually you will have to be more independent than when you started graduate school. The earlier you start on that path to independence the better. Independence will play a critical part in your career as an innovative scientist. As much as possible define your own research project with a view to make a significant and unique scientific contribution.

## Rule 4: Remember, Life Is All about Balance

Take the time to meet your own needs. Graduate school is highly demanding, both mentally and physically. Your health comes first, spend the time being healthy or else you might find yourself spending more time being sick. Hard work should be balanced with other activities that you enjoy and give you a break. These activities can often become important in your future scientific career. Collaborations sometimes start not because of a shared scientific interest initially, but because you share the same hobby or other interest.

## Rule 5: Think Ahead and Develop Your Professional Career Early

There are two parts to this. The first part relates to professional development. Being a successful scientist is more involved than just doing good science. You need to be able to write good papers, submit compelling scholarship and grant applications, make powerful presentations, and communicate and collaborate with other researchers. The other Ten Simple Rules editorials are a start here [1–7], but you need to work on developing these skills at the same time as you work on your thesis. The second part involves using these emergent skills to figure out what to do with the higher postgraduate degree. Do not wait until you graduate to take the next step. Have a position and a fellowship, if possible, lined up ahead of time.

## Rule 6: Remain Focused on Your Hypothesis While Avoiding Being Held Back

Formulation of the hypothesis is the first thing you'll learn in Science 101, and yet somehow it seems to get occasionally thrown out the window. When you find yourself lost in the details of your research, take a step back and remind yourself of the big picture. Revaluate your hypothesis from time to time to see if it still makes sense, because you may find yourself needing a new one. Always keep this in mind in discussions with your mentor. As you have these discussions, remember you are cheap labor, and, if you are a good student, a source of success to your mentor. The temptation is that your mentor will want to keep you around as long as possible. Define the scope of your project early with your mentor and agree that this is what you will attempt to complete in order to receive the degree. A career awaits you beyond the laboratory of your graduate student days. Do not prolong moving on to new challenges.

## Rule 7: Address Problems Earlier Rather Than Later

If graduate school wasn't quite what you thought it would be, be it scientifically or otherwise, find out what your options are to address the problem. Discuss these problems with your mentors. A good mentor is there not just to guide you scientifically, but also in your personal development. Remember, they have been there themselves and have likely seen similar issues with earlier students. Take time off to reflect on your future if this is needed. A good mentor will understand that you come first.

## Rule 8: Share Your Scientific Success with the World

Being recognized by your peers as someone who does good science is important both within your institution, nationally, and internationally. When opportunities arise to give seminars and presentations to other groups, take them. Before starting with a mentor, come to an agreement as to when and what meetings you can attend locally and globally. Scientific meetings are a fun and fruitful venue for exchange. Be sure to venture beyond the comfort zone of familiar faces, because it is important to meet other colleagues in your field. These people may become your future collaborators, friends, advocates, and employers.

## Rule 9: Build Confidence and a Thick Skin

As you pave the road to scientific fame with Rule 8, expect your work to be criticized and scoffed at, for that is part of the scientific process of challenging new ideas. The best way to build self-confidence for these otherwise defensive moments is to be prepared and to present your work clearly with a confident display of your expansive knowledgebase of the relevant related work. Do not be intimidated by big names who question your work; counter knowledge with knowledge. Another reason to have a thick skin is that the path to success will not be without setbacks—setbacks such as experiments that fail, and experiments that succeed but do not yield a useful result causing you to have wasted significant time. Undergraduate training is usually much more structured and does not prepare you for such setbacks. Learn as much as you can from these situations both about the science and yourself and move on.

## Rule 10: Help Select and Subsequently Engage Your Thesis Committee

This rule depends somewhat on how your institution is structured. Some institutions do not convene a thesis committee until near the end of your work. For those institutions that require a thesis committee to be convened early, talk with your mentor and be involved in the selection process. The committee is there to work for you as secondary mentors. Consider people whose own research experience will be valuable to you or who have a reputation for ongoing mentoring in all areas of professional development. Make a point of talking to members of the committee from time to time and keep them abreast of what you are doing. On occasion, you and your primary mentor may have disagreements; committee members can be invaluable here. 
